# Syphilis Screening among 27,150 Pregnant Women in South Chinese Rural Areas Using Point-of-Care Tests

**DOI:** 10.1371/journal.pone.0072149

**Published:** 2013-08-29

**Authors:** Li-Gang Yang, Joseph D. Tucker, Feng-Ying Liu, Xu-Qi Ren, Xuan Hong, Cheng Wang, Megan M. McLaughlin, Cedric H. Bien, Xiang-Sheng Chen, Bin Yang

**Affiliations:** 1 Division of STD Control, Guangdong Provincial Center for STI & Skin Diseases Control and Prevention, Guangzhou, China; 2 National Center for STD Control, Nanjing, China; UCL Institute of Child Health, University College London, United Kingdom

## Abstract

**Objectives:**

To determine the prevalence and correlates of syphilis among pregnant women in rural areas of South China.

**Methods:**

Point-of-care syphilis testing was provided at 71 health facilities in less developed, rural areas of Guangdong Province. Positive samples were confirmed at a local referral center by toluidine red unheated serum tests (TRUST) and *Treponema pallidum* particle agglutination (TPPA) tests.

**Results:**

Altogether 27,150 pregnant women in rural Guangdong were screened for syphilis. 106 (0.39%) syphilis cases were diagnosed, of which 78 (73.6%) received treatment for syphilis. Multivariate analysis revealed that older pregnant women (31–35 years old, aOR 2.7, 95% CI 0.99–7.32; older than 35 years old, aOR 5.9, 95% CI 2.13–16.34) and those with a history of adverse pregnant outcomes (aOR 3.64, 95% CI 2.30–5.76) were more likely to be infected with syphilis.

**Conclusions:**

A high prevalence of syphilis exists among pregnant women living in rural areas of South China. Enhanced integration of syphilis screening with other routine women's health services (OB GYN, family planning) may be useful for controlling China's syphilis epidemic.

## Introduction

Globally an estimated 1.36 million pregnant women had active syphilis in 2008 [1]. In that year, maternal syphilis resulted in more than 520,000 adverse outcomes, including stillbirths, early fetal losses, neonatal deaths, preterm or low birthweight infants, and infants born with congenital syphilis (CS) [1]. Many of these women seek prenatal care in rural regions of low- and middle-income nations that do not have the capacity to implement traditional syphilis screening [1]–[4]. In turn, pregnant women in these contexts either receive testing during the third trimester after their babies have been affected or never receive testing [1]–[3].

This lack of syphilis testing in rural regions leads to uncertainty about the extent of the syphilis epidemic among rural pregnant women. However, developments in point-of-care (POC) technology now allow accurate syphilis screening using immunochromatographic strips [5]. These tests, which have demonstrated sensitivity and specificity comparable to laboratory-based methods, can provide a rapid result within 30 minutes of testing. This development enables routine syphilis testing in low-level rural health facilities that serve large numbers of pregnant women [6].

Responding to the need for improved syphilis screening across our province in China, we launched a rural syphilis project to examine the prevalence and correlates of infection.

## Methods

### Ethics Statement

This study was approved by the GDSSC Institutional Review Board in Guangzhou, China. Informed consent was obtained from all subjects who agreed to participate in the study.

### Project site and medical settings

The prevalence of syphilis in China has increased during the past decade and it is now one of the most common infectious diseases in China, particularly in South China [7], [8]. In 2011, a total of 429,677 syphilis cases were reported in China, representing 32.0 cases per 100,000 individuals [9]. The southern province of Guangdong reported 48.9 syphilis cases per 100,000 individuals [10]. The Pearl River Delta (PRD) is the population-dense, urbanized central region of Guangdong province that includes seven of the province's 21 municipalities. Similar to our previous research [6], we selected health facilities in the province's 14 less developed municipalities outside the PRD to participate in this research study. One or two counties were selected in each municipality. In each county, we invited hygiene stations, women and children's hospitals, and general hospitals to participate in the study. Township hygiene stations are the most basic local public health institutions serving villagers in rural China. They offer primary care and preventive health services and are typically where women in rural China give birth.

A referral center, typically a local sexually transmitted disease (STD) control center, was designated in each study county. The referral centers were qualified to perform both nontreponemal tests (e.g. toluidine red unheated serum test, or TRUST) and treponemal tests (e.g. *Treponema pallidum* particle agglutination assay, or TPPA).

### Screening and referral

In each participating health facility, free rapid, POC syphilis testing (Acon Biotech Co. Ltd, Hangzhou, China) was provided to pregnant women during antenatal care after obtaining informed consent. They received the results of the rapid testing at the same visit. All positive sera were retested with both the TRUST (Rongsheng Bio-technology Limited Corporation, Shanghai, China) and TPPA (Fujirebio Inc, Japan) tests at the local referral center. The test results were sent back to the health facilities within three days, and a clinician called the patient to provide the confirmatory test results. A syphilis case was defined as both TRUST- and TPPA-positive. Free treatment with benzathine according to national STD management guideline was provided to syphilis cases at the local referral center. All staff involved in the study received a half-day training before the start of the research.

### Data collection and analysis

The medical staff who prescribed testing for the pregnant women completed a form that collected data on demographic characteristics, sex partners, and past gynecologic and obstetric history. Women ages 18 years or older were included in the study. The local referral center entered the data into Epidata and sent the compiled data to the Guangdong Provincial Center for STI & Skin Diseases Control (GDSSC) on a monthly basis. Univariate and multivariate logistic regression was used to identify factors associated with testing positive for syphilis. All variables examined in the univariate analysis were considered as candidates for the multivariate model. A backward elimination process was used to build the final multivariate model, and a p-value <0.05 was required for retention in the final model. SPSS 11.5 (Chicago, IL) was used for all analyses. This study was approved by the GDSSC Institutional Review Board in Guangzhou, China.

## Results

A total of 27,150 pregnant women in rural Guangdong received POC syphilis screening. They were recruited from 55 hygiene stations, 12 general hospitals, and 4 women and children's hospitals. Demographic characteristics of pregnant women who received screening are presented in Table 1. Women included in this study were primarily married (96.9%), middle school educated (73.8%), and between the ages of 21 and 30 years (72.6%). A large proportion of women in the study (43.8%) had no previous children. At the time of syphilis screening, only 17.3% of women were in their first trimester; most women (53.6%) were in their third trimester or later. Among the pregnant women screened, 106 (0.39%) were diagnosed with syphilis infection. Of these 106 cases, 78 (73.6%) received treatment for syphilis.

**Table 1:** Demographic characteristics of pregnant women screened for syphilis (N = 27,150).

Characteristic	No. of women screened	Proportion (%)
Age		
Less than 21 years old	2,699	9.9
21 to 25 years old	10,616	39.1
26 to 30 years old	9,108	33.5
31 to 35 years old	3,228	11.9
More than 35 years old	1,499	5.5
Marital status		
Single*	847	3.1
Married	26,303	96.9
No. of children		
Zero	11,883	43.8
One	10,574	38.9
Two or more	4,537	16.7
Highest level of school completed		
Illiterate/elementary school	2,637	9.7
Middle school	20,034	73.8
High school	3,462	12.8
More than high school	860	3.2
Gestational trimester at testing		
First trimester	4,699	17.3
Second trimester	5,041	18.6
Third trimester and later	15,294	56.3
Time spent residing in current community (months)		
Less than 3 months	2,710	10.0
3 to 6 months	989	3.6
6 to 12 months	1,315	4.8
More than 12 months	19,256	70.9

* Includes women who are not married, separated, widowed, and divorced.

Univariate analysis revealed a higher proportion of syphilis infection among older women, unmarried women, women with a history of adverse pregnancy outcome, and women whose partners spent between 1 week and 3 months outside the home (Table 2). Compared to women 20 years or younger, women ages 31 to 35 and women ages 31 to 35 were significantly more likely to be infected with syphilis (odds ratio [OR] 3.19, 95% confidence interval [CI] 1.19–8.56; OR 7.29, 95% CI 2.73–19.46, respectively). Unmarried women were 2.55 times more likely to be infected (95% CI 1.24–5.26) compared to married women. A history of adverse pregnancy was associated with 3.81 times greater odds of syphilis infection (95% CI 2.50–5.81). And compared to women whose partners spent less than 1 week outside the home, those whose partners spent 1 week to 3 months outside the home were more likely to be infected (OR 3.03, 95% CI 1.45–6.32).

**Table 2:** Univariate and multivariate analysis of demographic and socioeconomic factors associated with positive syphilis test among pregnant women.

	Positive syphilis test		Univariate analysis		Multivariate analysis	
Characteristic	No. positive (n = 106)	% positive (0.4%)	OR (95% CI)	*p*	OR (95% CI)	*p*
Age						
Less than 21 years old	5	0.2	1.00		1.00	
21 to 25 years old	24	0.2	1.22 (0.47–3.20)	0.69	1.01 (0.38–2.68)	0.99
26 to 30 years old	38	0.4	2.26 (0.89–5.74)	0.09	1.81 (0.71–4.67)	0.22
31 to 35 years old	19	0.6	3.19 (1.19–8.56)	0.02	2.70 (0.99–7.32)	0.05
More than 35 years old	20	1.3	7.29 (2.73–19.46)	<0.01	5.90 (2.13–16.34)	<0.01
Marital status						
Single*	8	0.9	2.55 (1.24–5.26)	0.01		
Married	98	0.4	1.00			
Highest level of school completed						
Illiterate/elementary school	19	0.7	6.23 (0.83–46.64)	0.08		
Middle school	73	0.4	3.14 (0.44–22.63)	0.26		
High school	13	0.4	3.24 (0.42–24.29)	0.26		
More than high school	1	0.1	1.00			
No. of children						
Zero	25	0.2	1.00			
One	49	0.5	2.21 (1.36–3.58)	<0.01		
Two or more	32	0.7	3.37 (2.00–5.70)	<0.01		
History of adverse pregnancy outcome						
No	74	0.3	1.00			
Yes	31	1.2	3.81 (2.50–5.81)	<0.01	3.64 (2.30–5.76)	<0.01
Gestational trimester at testing						
First trimester	17	0.4	1.00			
Second trimester	26	0.5	1.43 (0.77–2.64)	0.25		
Third trimester and later	47	0.3	0.85 (0.49–1.48)	0.56		
Time spent residing in current community (months)						
Less than 3 months	10	0.4	1.00			
3 to 6 months	4	0.4	1.10 (0.34–3.50)	0.88		
6 to 12 months	8	0.6	1.65 (0.65–4.20)	0.29		
More than 12 months	76	0.4	1.07 (0.55–2.07)	0.84		
Amount of time husband/boyfriend slept outside the house in last 12 months						
Less than 1 week	71	0.4	1.00			
1 week to 1 month	11	0.6	1.79 (0.95–3.39)	0.07		
1 to 3 months	8	1.1	3.03 (1.45–6.32)	<0.01		
3 to 6 months	3	0.7	2.08 (0.65–6.63)	0.22		
More than 6 months	12	0.3	0.92 (0.50–1.70)	0.78		

* Includes women who are not married, separated, widowed, and divorced.

In the multivariate analysis, older age and history of adverse pregnancy outcome remained significantly associated with syphilis infection (Table 2). Compared to women less than 21 years old, women 31–35 years of age were 2.70 times more likely to be infected with syphilis (95% CI 0.99–7.32), and women older than 35 years were 5.90 times more likely to be infected (95% CI 2.13–16.34). Women with a history of adverse pregnancy outcomes were 3.64 times more likely to have syphilis (95% CI 2.30–5.76) compared to those without a history of adverse outcomes.

## Discussion

Syphilis remains a major cause of preventable death and disability among neonates and young children [3], [11], [12]. Although an estimated 80% of pregnant women with active syphilis attend antenatal care, many of these women are not tested for syphilis [1]. Approximately 60% of the more than 520,000 adverse outcomes caused by maternal syphilis occur among women who attend ANC but are not tested or treated for syphilis [1]. To our knowledge, this is the first comprehensive syphilis study conducted among pregnant women in rural China. China has experienced a resurgence of syphilis during the past 20 years [13]. One population-representative survey [14] and case report data suggest [7] that urban Chinese have more sexual risk behaviors and a greater burden of STIs [13]. However, syphilis diagnostic capacity in rural health facilities is limited [6], and there have been few studies of syphilis outside of urban areas [13], [15]. Thus, the burden of syphilis in rural areas is poorly understood. Additionally, in 2003–2004 the Chinese government phased out the compulsory premarital health check that included syphilis screening [13], and there have been few comprehensive studies of the syphilis burden in non-core groups since then. This study sought to address these gaps by examining the prevalence of syphilis among pregnant women accessing care at health facilities in rural Guangdong province.

Our study found a high burden of syphilis among rural pregnant women in Guangdong Province. The overall syphilis prevalence (0.39%) among pregnant women in this study is comparable to the prevalence reported among pregnant women in urban Fuzhou (0.2%), Shanghai (0.3%), and Shenzhen (0.5%) [16]–[18]. The burden of syphilis among this population of pregnant women in rural South China is also similar to what has been reported in studies conducted at regional blood donation centers that cover urban and rural areas throughout China (0.30–0.63%) [19]–[21]. Compared to other countries, the prevalence reported here is lower than among pregnant women in rural low-income settings in sub-Saharan Africa and Haiti [22]–[30] but higher than among women in rural areas in high-income countries like the U.S. [31] The syphilis epidemic among pregnant women in rural Guangdong province might be partially explained by female migrants or their husbands returning to their rural origins from the cities with untreated syphilis and persistently risky sexual behavior [32]. Studies have found that migrants in some Chinese cities have high-risk sexual behavior [33] and a higher prevalence of syphilis compared to local residents [16], [17], but few studies have examined the sexual risk of returning migrants.

We found that older age was associated with syphilis infection among pregnant women in rural Guangdong province. National STI surveillance data show that men over 50 years of age represent a substantial and increasing proportion of notified syphilis cases in China [34]. However, it is not clear whether these patterns reflect an increased sexual risk in this population or an increased likelihood of being routinely screened as part of a hospital admission. A national population-based study also demonstrated that chlamydia infection was associated with older age among women [14]. These results are consistent findings from sub-Saharan Africa, Australia, and the U.S. that also reveal a sizable burden of STIs among older persons [35]. Additionally, a study of STI patients in Guangxi showed high rates of purchasing commercial sex and low rates of condom use in individuals 50 years and older [34], [36]. Our finding that older women were more likely to be infected with syphilis suggests the need to integrate syphilis screening with routine health services for older women, such as screening for breast, cervical, and other cancers. Taking advantage of these opportunities to establish periodic syphilis screening is an important way of reaching women who are no longer having pregnancies and thus not accessing screening offered during antenatal care.

Women with a history of adverse pregnancy outcomes were also more likely to be infected with syphilis in this study. This finding is consistent with the observation that approximately 60% of pregnancies in women with untreated syphilis will result in some adverse outcome [11]. About 40% of such pregnancies will end in miscarriage, stillbirth, or perinatal death, and surviving newborns are at risk for congenital malformations [11], [37]–[39]. The higher prevalence of syphilis among women with a history of adverse pregnancy outcomes also points to an opportunity for integrating syphilis control and reproductive health services beyond antenatal care. Few studies of STIs in China have examined women seeking termination of pregnancy, but this population may be in particular need of STI screening because unsafe sexual behavior is a risk factor for both unwanted pregnancy and STIs [40]. Integrating syphilis screening with family planning services, such as intrauterine device insertions, also represents another way of reaching older women. HIV testing has been successfully integrated with post-abortion and other family planning services in settings in Kenya [41], Ethiopia [42], Tanzania [43], and the U.S. [44]

This study revealed a high burden of syphilis in rural South China among a non-core group believed to be at low risk. These findings suggest that syphilis may be moving toward a generalized epidemic in China. Making syphilis testing universally available in rural health facilities is fundamental in order to respond this challenge. The 10-year National Syphilis Prevention and Control Plan, announced by the Ministry of Health in 2010, is a promising step forward with its emphasis on expanding syphilis testing coverage among pregnant women in urban and rural areas [45]. However, the screening and treatment coverage targets are too low to meet the goal of reducing congenital syphilis by 2020 [46]. A key element missing from the national plan is an emphasis on earlier antenatal screening. Mother-to-child transmission of syphilis is preventable if the mother receives adequate treatment early during the pregnancy, ideally during the first trimester. In our study, more than half of the pregnant women had their first syphilis screening in the third trimester or around the time of delivery, some even after labor. This is consistent with a study in a neighboring province in South China that found that 57.7% pregnant women in rural areas had not yet made their first antenatal visit by the last month before labor [47]. Thus, improving antenatal care coverage is key for effective syphilis control.

This study has a number of limitations. We cannot confirm whether adverse pregnancy outcomes reported by women with syphilis infection were syphilis-related. Some of these adverse outcomes may have predated the syphilis infection detected in this study. Additionally, our data on linkage to care may underestimate the proportion of syphilis cases that received treatment if patients sought treatment in locations other than the local referral center, such as private health care facilities. Despite these limitations, this study offers valuable data on the prevalence of syphilis among a large population of pregnant women throughout rural areas of Guangdong province.

To achieve the goals of the National Syphilis Prevention and Control Plan, the capacity for diagnosing syphilis in rural health facilities in China needs to be strengthened. Rapid POC syphilis testing, which has been used in remote communities in Brazil [48] and other settings [5], is an excellent option, especially in rural areas with limited laboratory facilities. In our project, syphilis screening by POC syphilis testing with referral of positive cases to local STD centers was well accepted by rural pregnant women and local health facilities. Improving antenatal care coverage, promoting retention in care, and integrating syphilis screening with other routine health services are also key for effective syphilis control in rural China.
